# The suppressive potential of a gene drive in populations of invasive social wasps is currently limited

**DOI:** 10.1038/s41598-023-28867-8

**Published:** 2023-01-30

**Authors:** Adriaan B. Meiborg, Nicky R. Faber, Benjamin A. Taylor, Brock A. Harpur, Gregor Gorjanc

**Affiliations:** 1 HighlanderLab, The Roslin Institute and Royal (Dick) School of Veterinary Studies, The University of Edinburgh, Easter Bush Campus, Midlothian, EH25 9RG UK; 2 Developmental Biology Unit, European Molecular Biology Laboratory, Meyerhofstraße 1, 69117 Heidelberg, Germany; 3 Laboratory of Genetics, Department of Plant Sciences, Wageningen University & Research, Droevendaalsesteeg 1, 6708 PB Wageningen, The Netherlands; 4 Department of Entomology, Purdue University, West Lafayette, IN 47907 USA

**Keywords:** Computational biology and bioinformatics, Computational models

## Abstract

Social insects are very successful invasive species, and the continued increase of global trade and transportation has exacerbated this problem. The yellow-legged hornet, *Vespa velutina nigrithorax* (henceforth Asian hornet), is drastically expanding its range in Western Europe. As an apex insect predator, this hornet poses a serious threat to the honey bee industry and endemic pollinators. Current suppression methods have proven too inefficient and expensive to limit its spread. Gene drives might be an effective tool to control this species, but their use has not yet been thoroughly investigated in social insects. Here, we built a model that matches the hornet’s life history and modelled the effect of different gene drive scenarios on an established invasive population. To test the broader applicability and sensitivity of the model, we also incorporated the invasive European paper wasp *Polistes dominula*. We find that, due to the haplodiploidy of social hymenopterans, only a gene drive targeting female fertility is promising for population control. Our results show that although a gene drive can suppress a social wasp population, it can only do so under fairly stringent gene drive-specific conditions. This is due to a combination of two factors: first, the large number of surviving offspring that social wasp colonies produce make it possible that, even with very limited formation of resistance alleles, such alleles can quickly spread and rescue the population. Second, due to social wasp life history, infertile individuals do not compete with fertile ones, allowing fertile individuals to maintain a large population size even when drive alleles are widespread. Nevertheless, continued improvements in gene drive technology may make it a promising method for the control of invasive social insects in the future.

## Introduction

Invasive species represent a global issue that has worsened with increased global trade and transportation^1,2^. Suppression of these invasive species is often prohibitively expensive, labour intensive, and largely ineffective^1^. One such species currently invasive in Europe is the yellow-legged hornet *Vespa velutina nigrithorax*, hereafter called the Asian hornet. This insect was introduced to France from Southern China in 2004^3,4^ and quickly spread to the whole of France, most of the Atlantic coast, Northern Italy, Belgium, parts of the United Kingdom, and parts of Germany, where the northernmost finding was made in 2020^5,6^. This spread is in accordance with previous modelling of suitable environments^7,8^. Modelling also showed that there are many more areas in Europe suitable for the Asian hornet to invade^8^.

The Asian hornet likely has a serious impact on commercial bee colonies^9–11^ and potentially on other pollinators such as wild bees and syrphids^5,11^. Up to two thirds of its diet consists of honey bees^11^, and the annual loss for France’s honey and pollination industry in 2015 was estimated at 53.3 million euros^8^. The invasion probably started with only a single fertilised queen^3^, which underscores the great invasive potential of the Asian hornet. Indeed, one queen produces on average 300 gynes, which are reproductive female offspring, that all have the potential to start a new nest the next year^12^. On top of this great reproductive potential, controlling the Asian hornet through conventional means is difficult; the nests are high up in trees, and thus hard to find, and they are also hazardous to approach. Bait trapping with food or chemicals is currently the most reliable control method, though it is only partially effective and is not species-specific^13^. These inadequacies highlight the need to find an effective strategy to control the Asian hornet before it spreads to other suitable regions.

Over the last decade, gene drives have emerged as a potential tool to control invasive populations for which other measures are ineffective^14–18^. A gene drive is a genetic element that spreads through a population over generations at a super-Mendelian rate. For population control, it is engineered to impose a fitness cost once it is prevalent. For example, a gene drive may disrupt a haplo-sufficient female fertility gene. Haplo-sufficient means that a single functioning copy of the gene is enough for a female to be fully fertile. At first, the gene drive is present mostly in a heterozygous state due to matings with wildtype individuals. Once it reaches a higher frequency, matings between gene drive individuals will occur more frequently and offspring will be homozygous for the gene drive, with female offspring thus being infertile. This way, population fecundity declines through the reduced fertility of homozygous individuals^15^. Gene drives have been demonstrated to work in yeast^19^, fruit flies^20^, mosquitoes^21,22^, and mice to a lesser extent^23,24^. The field has recently focused on improving safety and containment which would make gene drives controlled enough for release in the wild^25^. A range of gene drives have been designed to be less invasive by nature, or to mitigate risks to an extent. Some stop spreading after a certain number of generations and are thus self-limiting^26–28^, some require high introduction frequencies^29–31^, some can stop or remove a gene drive that is already present in a population^32,33^, and some target locally fixed alleles so that the gene drive cannot spread in non-target populations^34^. With the advance of such containable gene drives, we can start to consider gene drive technology as a potential tool for controlling invasive social insects like the Asian hornet.

Creating a realistic life history model for a population is one of the first steps in determining if a gene drive might be an effective control agent^35^. A previous study has modelled a gene drive causing male sterility in another haplodiploid social hymenopteran, the common wasp *Vespula vulgaris*, that is invasive in New Zealand^36^. This type of gene drive was shown to be only mildly promising, because there was a trade-off between the spread and the impact of the gene drive. Namely, if the gene drive causes complete male sterility, it is unlikely to spread, whereas if the gene drive causes incomplete male sterility, it is unable to impact the fertility sufficiently for population control^36^. As such, this specific gene drive design works similarly to sterile insect technology: a powerful, but only temporary method. Therefore, other gene drive designs are needed to control such populations more efficiently^37^. For example, three recent modelling studies showed that gene drives can work in haplodiploid species under specific conditions^38–40^.

In this study, we model several gene drive strategies to investigate the potential of gene drive technology to control Asian hornet populations. We look into both gene drive parameters and life history traits to find the most important factors that could affect the success of gene drives to control these invasive populations. We developed a model that can be easily adapted to other social haplodiploid insects, as the Asian hornet is not the only invasive social hymenopteran. Indeed, this group of insects, which is comprised of wasps, bees, sawflies and ants, contains many successful invasive species^41^. We demonstrate the flexibility of the model by modelling a second invasive social hymenopteran, *Polistes dominula*, hereafter called the European paper wasp. This paper wasp is a widespread invasive species with a very different biology than the Asian hornet. It has much smaller colonies, and queens are almost exclusively monogamous^42^. Our results show that gene drives can be used to suppress an invasive population of either species, but only when the gene drive achieves high efficiency. This is the case because of a combination of two reasons: high per-colony surviving offspring numbers, and the social wasp life history that prevents infertile individuals from competing with fertile ones. Overall, because the required drive efficiency indicated by our models is close to the technological limits of existing synthetic drives, our results suggest that extensive testing will likely be required before this technology can be applied to suppress populations of invasive social wasps.

## Materials and methods

**Figure 1:**
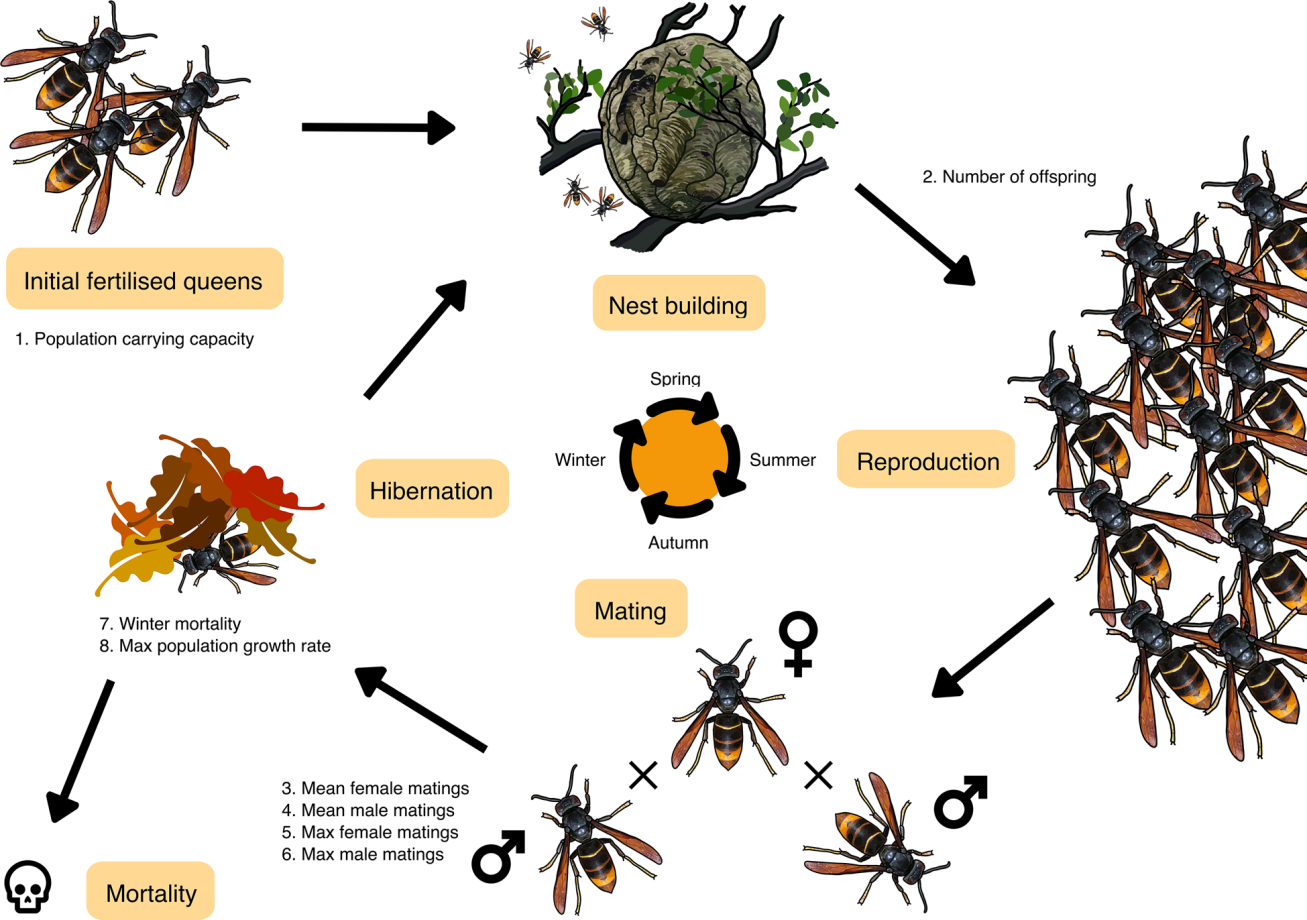
Overview of the demographic model. For a specific explanation of the model, see the “Materials and methods” section.

The model is an individual-based, stochastic, year-by-year model with two main parts: (1) a realistic model of a social wasp population based on their life history and (2) the implementation of a gene drive (see Fig. 1). The model was built using R version 4.1.2^43^. We use AlphaSimR package version 1.0.4^44^. This package is designed for animal and plant breeding studies, but also serves as an excellent tool for modeling population genetics studies. The model can be found on GitHub at https://github.com/HighlanderLab/ymeiborg_hornet_gd.

### Model structure

The structure of our model mimics the life cycle of a typical haplodiploid social wasp. Throughout the model, we only track female numbers and genotypes, as males only live for a short amount of time for mating purposes and little is known about their survival. We initiate the model with an equal number of females and males, of which the females (queens) represent the starting population size. Since we initiate the model with mated queens, each male is assigned as a mate for a queen. Depending on the chosen number of generations to model, the following steps are repeated:

1. **Offspring generation**. First, offspring are generated for each successfully mated queen. This is the point at which gene drive dynamics, such as homing or resistance allele formation, take place in the queen’s germline as described in “Gene drive implementation” section. Haploid male offspring are produced by the queen from unfertilised eggs, while diploid female offspring originate from fertilised eggs. For each female that has mated more than once, the total number of offspring is divided randomly over the multiple mates. We assume an equal number of female and male offspring. Previous findings report a 3:1 male to female ratio in invasive populations^45^, but this is likely a result of overproducing infertile diploid males under inbreeding^46^, as non-Fisherian sex ratios are unlikely to persist at a population level^47,48^. As diploid males are sterile and thus do not contribute to the population further, we have decided to use a standard 1:1 sex ratio instead. If there were a lot of sterile males, this might cause females to mate less successfully, but since we are using a maximum population growth rate based on observational data from an invasive populations, we believe this influence on the outcomes of our model to be minimal. The number of female and male offspring are used as averages for a Poisson distribution for each sex so there is natural variation in the number of female and male offspring from each queen. For Asian hornets, on average, one queen produces around 300 females (gynes)^12^ and we assume the same number for males. European paper wasp nests are much less productive, with a single nest producing ~ 20 offspring of each sex^49^.
2. **Mating**. We have implemented random mating between males and females, except when we impose a fitness costs of having a gene drive here. Both males and females with a gene drive in their genome then have a certain probability of being removed from the mating pool. We assumed full dominance for the fitness cost, so both heterozygous or homozygous individuals are equally affected. Since we are modelling a haplodiploid species, males can only be hemizygous for the gene drive, and females will be infertile when homozygous for the gene drive anyway, so this way the fitness cost will impact males and females similarly.
  Next, as polyandry is shared by many social hymenoptera^50,51^, we have included it in the model. For both females and males, the model takes the mean number of matings, the minimum number of matings, the maximum number of matings, and based on these values generates a random number of matings from a truncated Poisson distribution. For males, the mating frequencies from Ross^52^ include a high frequency of non-mating males, so we have not zero-truncated their mating rate distribution.
  The Asian hornet queen is polyandrous and is able to attract mates at great distances^53^, so for mating rate we use a zero- and max-truncated Poisson distribution with a mean of 3.275 and a maximum of 4^3^. Male Asian hornet mating rates are unknown, so we used a max-truncated Poisson distribution with a mean of 0.9 and a maximum of 3 based on mating frequencies for *Vespula maculifrons*^52^. Mating rates for European paper wasp females are estimated to fall between 1 and 1.1^54,55^, and as such were modeled using a zero- and max-truncated Poisson distribution with a mean of 0.2 and a maximum of 2. There is no accurate data that capture total male reproductive output in *P. dominula* either, so we used the rates reported in *V. maculifrons*^52^ instead.
  We assume that neither female or male knows whether their mate is fertile until the offspring generation next year, at which point it is too late to find a new mate. This assumption is in line with the observation that hymenopteran females are largely unable to discriminate against infertile but otherwise healthy diploid males^46^.
3. **Winter mortality**. After mates have randomly been selected this way, we subject the female population to winter mortality. There is no mortality data available for the Asian Hornet so we used the value of 0.97 estimated from *Vespula vulgaris* which has a very similar life history^56^. Data on *Vespa affinis*, a hornet overlapping with the Asian hornet’s native range, suggest high winter mortality rates are likely, as *V. affinis* queens generally die within 24 h after emergence for hibernation. Winter mortality data for the European paper wasp is more readily available, and suggest a mortality rate of about 0.5^57,58^. Mortality is applied uniformly across the female population with no consideration for genotypes.
4. **Densitiy dependent mortality**. When the queens who survive hibernation emerge, we remove those who are infertile or who have mated with infertile males. We remove these pairing before density dependent mortality sets in, as we assume they will fail to build a primary nest and fail to compete with the other foundresses for resources. Note that in the context of gene drive dynamics, density dependence is important because it will significantly impact gene drive performance, as reviewed by Dhole et al.^59^. The reproductive population size is regulated using a Poisson distribution around a logistic function, which ensures a maximum population growth rate and carrying capacity^60^:
  1 $$\begin{aligned} N_t = Poisson\left( \frac{K}{1+\frac{K-N_{t-1}}{N_{t-1}}*{r_{max}}^{-1}}\right) \text {,} \end{aligned}$$
  where $N_t$ is the number of females in generation *t*, $N_{t-1}$ the number of females in generation ${t-1}$, *K* is the carrying capacity, and $r_{max}$ is the maximum growth rate.
  For the Asian hornet, we use a maximum growth rate of 10^60^, and since there is no data available for the European paper wasp, we have used the same value of 10 for the maximum growth rate for them as well. Note that the maximum growth rate $r_{max}$ is calculated using $\frac{ln(R_0)}{T}$ with $R_0$ being the intrinsic growth rate observed by Franklin et al.^60^, *T* being the generation time of 1 year.
  Then, using the maximum possible population size in this generation and total number of female offspring, we calculate the mortality rate:
  2 $$\begin{aligned} P_{mortality} = 1-\frac{N_t}{\text {female offspring}}\text {.} \end{aligned}$$
  Each female thus has a probability of dying. The surviving females become queens and generate the offspring for the next generation. Male population numbers are not monitored, as they only live for a short amount of time and little is known about their survival.

### Gene drive implementation

Although AlphaSimR was designed to model large numbers of loci for quantitative genetics in plant and animal breeding, the framework is perfect for tracking the single locus of a gene drive. Each individual is modelled with a single gene drive locus and inheritance is random following Mendelian laws. We have implemented a basic homing gene drive which copies itself to the other chromosome in the germ line and has four potential alleles: wildtype (WT), gene drive (GD), resistance (RE), and non-functional (NF). We model diploid females and haploid males, so there is no gene drive activity in the male genome. We use three parameters in our gene drive implementation: a probability of cutting ($P_{cut}$, default 0.95^22^), a probability of non-homologous end-joining ($P_{NHEJ}$, default 0.02^22^), and a probability that functional repair occurs ($P_{FR}$, default 0.01^61,62^), which influences the rate at which resistance alleles form. Resistance alleles are formed when, following the DNA break made by the Cas9 protein, the DNA strand is repaired via non-homologous end-joining and the function of the gene is restored. Some repairs that restore the function of the gene will not consist of the same bases as the original gene due to the redundancy of the genetic code. The Cas9 guide RNA can now no longer cut the DNA sequence. The chance of this happening is called the probability of functional repair $(P_{FR})$.

Thus, if cutting, non-homologous end-joining, and functional repair occur, a resistance allele is created that is fully functional and cannot be cut and homed into by the gene drive. We evaluate the sensitivity of results to the default probability of cutting, probability of non-homologous end-joining, and probability of functional repair. We also model a fitness cost of carrying the gene drive abstracted as a certain probability of mortality before mating ($P_{HetMort}$, default 0^63^), which we also evaluate in the sensitivity analysis. We conservatively assume that the fitness cost of carrying the gene drive has complete dominance, that is, it is equally deleterious for homo-, hemi-, and heterozygotes.

In the scenario of releasing females with a gene drive, these are added at the start of the model when the population of mated queens is initiated. In the scenario of releasing males with a gene drive, these are added instead in the mating step in the first generation of the model. These release strategies most likely represent the way a gene drive release could occur in reality. Note that as a result, the gene drive introduction frequency is much lower in a male release than a female release (on average, 100 gene drives per 1000 mated females, versus 100 gene drives per 1000 times 300 males).

### Modelled scenarios

To show two sides of the social wasp spectrum, we do all our modelling for two species of invasive social wasps that have distinct life histories. The first species we model is the Asian hornet, which is an extremely successful invasive species, probably due to its ability to produce many new queens each generation. Females of this species are also polyandrous. The second species is the European paper wasp, which has a more modest number of offspring and females are rarely polyandrous.

We first focus on which type of homing gene drive might work best for social wasps. We test a neutral gene drive, which is a gene drive that does not impose any fitness costs (potentially carrying a fitness neutral cargo), and gene drives that cause female, male, and both-sex infertility. In these scenarios, we model current standard gene drive efficiencies: $P_{cut}$ = 0.95, $P_{FR}$ = 0.01, $P_{NHEJ}$ = 0.02, and $P_{HetMort}$ = 0, as mentioned above. Second, we vary the parameters of the gene drive to find the space in which a gene drive works in our species. First, we vary $P_{FR}$ and $P_{NHEJ}$ together, second we vary $P_{HetMort}$ and $P_{cut}$ together. Third, we vary life history parameters of the wasps to evaluate how these parameters influence gene drive performance. We used the same default values as before, namely $P_{cut}$ = 0.95, $P_{FR}$ = 0.01, $P_{NHEJ}$ = 0.02, and $P_{HetMort}$ = 0. Finally, as polyandry is a trait dependent on varying multiple life history traits together and is known to impact drive performance^64^, we test all of the gene drives designed in the first scenario with and without polyandry in both species using the same default values as described previously.

**Figure 2:**
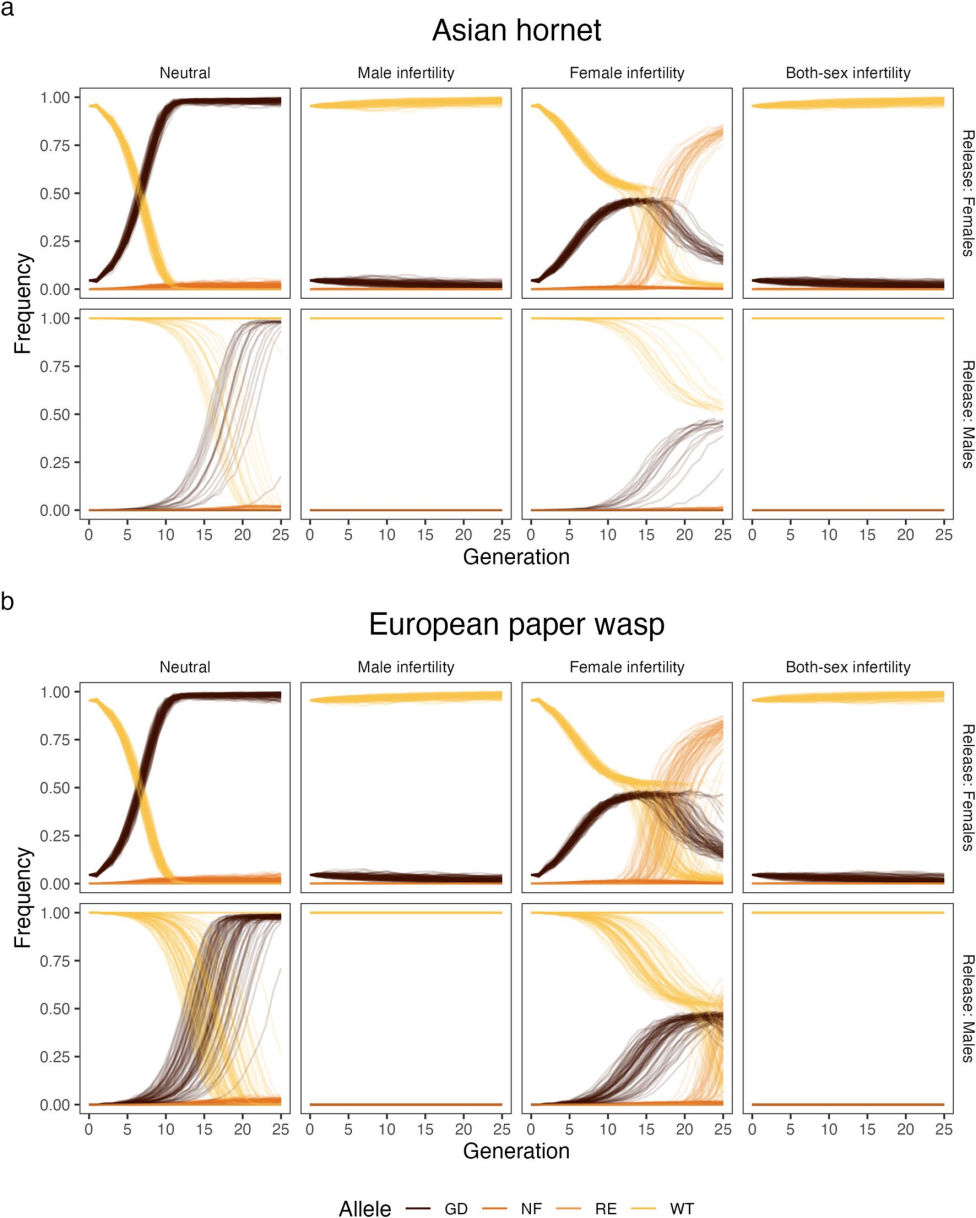
Frequencies of wildtype (WT), gene drive (GD), resistance (RE), and non-functional (NF) alleles in a female Asian hornet population (**a**) and a female European paper wasp population (**b**) by gene drive strategy and release gene drive carriers (females or males). The different strategies (neutral, male infertility, female infertility, and both-sex infertility) determine how the gene drive operates. In the neutral strategy there is no fitness cost to having the gene drive, whereas in the infertility strategies, the designated sex cannot reproduce when homo- or hemizygous for the gene drive.

For each model, except Fig. 2, Supplementary Figs. S1 and S2, we ran 10 replicates to get an estimate of the variance of the results. For Fig. 2, Supplementary Figs. S1 and S3, we ran 100 repetitions because the male gene drive release showed a lot of variation.

## Results

The spread of the gene drive depends heavily on its design. In the following paragraph we describe the frequencies of wildtype and gene drive alleles shown in Fig. 2, while population sizes for the same scenarios are available in Supplementary Fig. S1. A neutral gene drive spreads quickly through the female Asian hornet population, but does not always reach complete fixation as resistance alleles and non-functional alleles appear (Fig. 2a). These resistance alleles and non-functional alleles are then subject to random drift. A female release is much more effective in spreading the gene drive (Fig. 2a-top row) than a male release (Fig. 2a-bottom row). Although the gene drive reaches fixation in some cases, males fail to spread the gene drive in most cases. Moreover, the time to fixation is much more variable with male carriers. This is due to the lower introduction frequency of the gene drive in male releases as described in the methods.

Gene drives designed for population suppression show similar dynamics. When the gene drive targets fertility, we see that targeting male fertility prevented spread of the gene drive through the population. The spread stops immediately when the gene drive is introduced via males. This effect persists when targeting female fertility at the same time. A gene drive targeting female fertility after a female release spreads rapidly through the population, and leads to the suppression of the population in 64 cases out of 100. In the other 36 cases, resistance alleles form due to functional repair, and overtake the gene drive. When using a male release for female infertility, suppression occurred only 4 times out of 100 replicates.

There are key differences between the spread of the gene drive in the European paper wasp (Fig. 2b) and the Asian Hornet (Fig. 2a). As in the Asian hornet, female releases are more efficient, and female fertility is a better target for the gene drive. However, gene drive releases using males are more successful for the paper wasp than for the Asian hornet. This difference is due to the number of males in the mating swarm being smaller in the paper wasps, and thus the frequency of gene drives higher, as we have kept the number of introduced individuals the same between the two species. When targeting female fertility (Fig. 2b third column), the gene drive does rapidly reach near fixation as in the hornets, but suppression is less likely to occur, as only 20 out of 100 replicates ended in suppression (also see Supplementary Fig. S1). In male releases, only 6 repetitions out of 100 resulted in suppression.

For both species, the gene drive has no effect on the population size until complete suppression occurs (Supplementary Fig. S1). The gene drive is unable to partially suppress the population due to the reproductive strategy of the social wasps. Namely, infertile individuals are unable to generate workers and thus, we assume they do not compete for resources with fertile individuals. Furthermore, at equilibrium gene drive frequency, enough wildtype and heterozygote individuals are in the population to maintain the carrying capacity. A population is only suppressed if, by chance, all fertile queens die due to winter mortality or do not mate with a fertile male.

Gene drives targeting fertility are not able to reliably suppress the population in any of the modelled scenarios (Fig. 2, Supplementary Fig. S1). This is because resistance alleles rapidly spread through the population due to the heavy selective pressure the gene drive introduces. This way, these resistance alleles outcompete the gene drive, and rescue the population.

**Figure 3:**
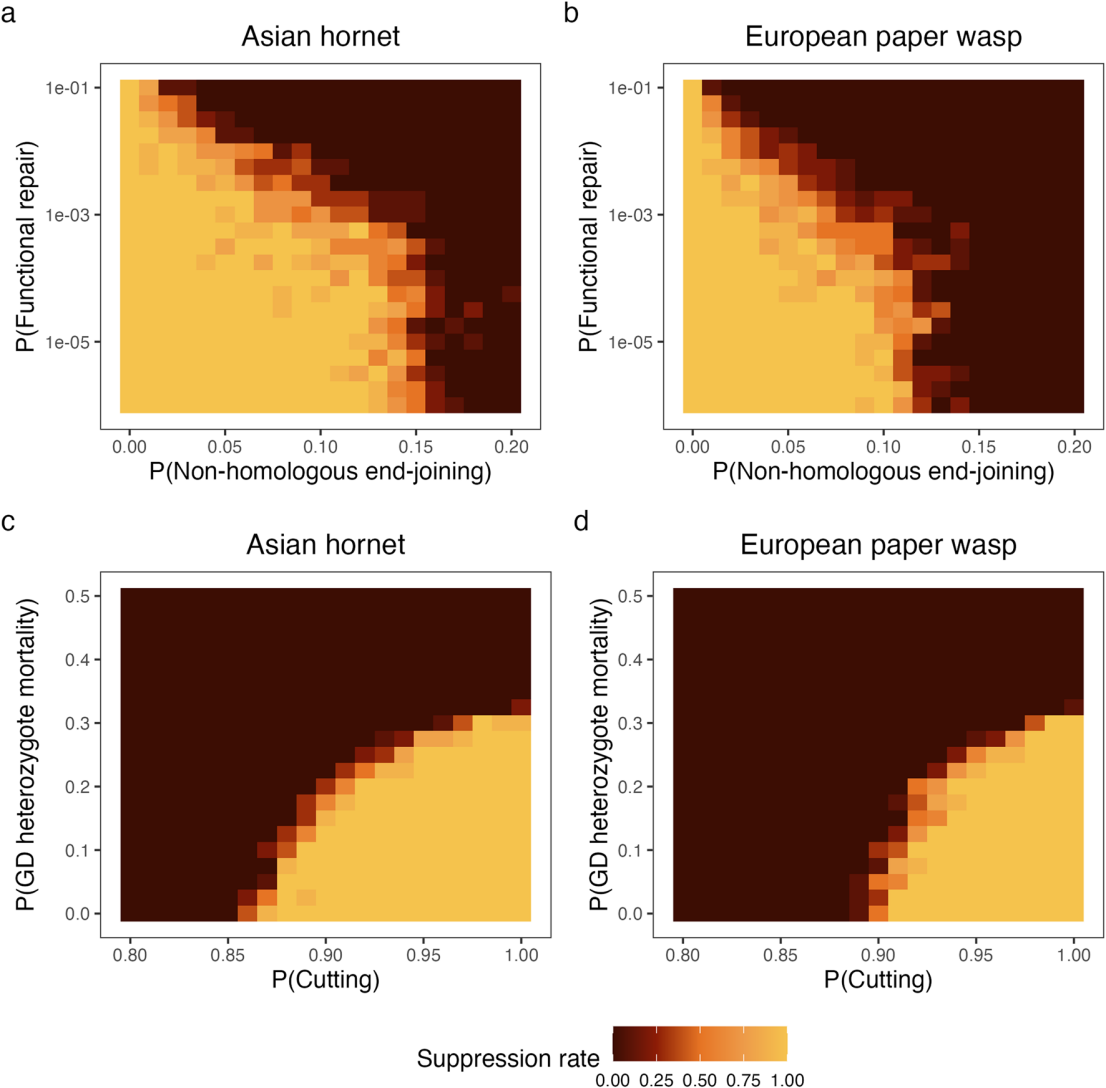
Heatmaps of the suppression rate in the Asian hornet (**a,c**) and the European paper wasp (**b,d**) using gene drives that have varying probabilities of functional repair ($P_{FR}$) and non-homologous end-joining ($P_{NHEJ}$), or cutting ($P_{cut}$) and mortality of gene drive heterozygotes ($P_{HetMort}$). Note that the model was run for 50 generations instead of 25 to reduce the noise and better allow the different scenarios to resolve.

Besides the formation of resistance alleles, the success of a gene drive is dependent on several other stochastic parameters: $(P_{FR})$, $(P_{NHEJ})$, $(P_{cut})$, and $(P_{mort})$. We varied these parameters and estimated which parameters are critical and what values are required for population suppression (Fig. 3, Supplementary Fig. S2). We only varied the parameters shown on the heat map, and kept the other ones at optimal values ($(P_{FR})$ = 0, $(P_{NHEJ})$ = 0, $(P_{cut})$ = 1, and $(P_{mort})$ = 0). Only when modelling one of $(P_{FR})$ or $P_{NHEJ}$, the other parameter cannot be set to its optimal value as that would negate the effect of the varied parameter. In these cases we used the default settings of $(P_{FR})$ = 0.01 or $P_{NHEJ}$ = 0.02.

In all modelled scenarios, the gene drive performs slightly worse in the European paper wasp (Fig. 3b,d, Supplementary Fig. S2b,d,f,h) than in the Asian hornet (Fig. 3a,c, Supplementary Fig. S2a,c,e,g). In both the Asian hornet and the European paper wasp, the probability of non-homologous end-joining is the most determining parameter together with the probability of functional repair. Only with extremely low values of $P_{FR}$ can we increase $P_{NHEJ}$ up to 0.12 in the Asian hornet and 0.08 in the European paper wasps (Fig. 3a,b, Supplementary Fig. S2a–d). The probability of gene drive heterozygote mortality is also quite similar between species (Fig. 3c,d, Supplementary Fig. S2c,d,g,h), with an absolute maximum being $P_{HetMort}$ = 0.3. Under optimal conditions, $P_{cut}$ can be lowered to 0.88 in the Asian hornet (Fig. 3c, Supplementary Fig. S2c) but only to 0.91 in the European paper wasp (Fig. 3d, Supplementary Fig. S2d).

The difference in gene drive efficiency between both species shows that life history traits influence the efficacy of the gene drive even between species with roughly similar life histories. To understand which life history parameters influence the efficacy of a suppression gene drive the most, we performed a sensitivity analysis on the following parameters from our model: max growth rate, mean female and male mating rate, max female and male mating rate, mean number of progeny, and winter mortality. To make sure the selected parameters actually show an effect of the gene drive, we also ran the population model without a gene drive to indicate which parameters result in a biologically unsustainable populations.

**Figure 4:**
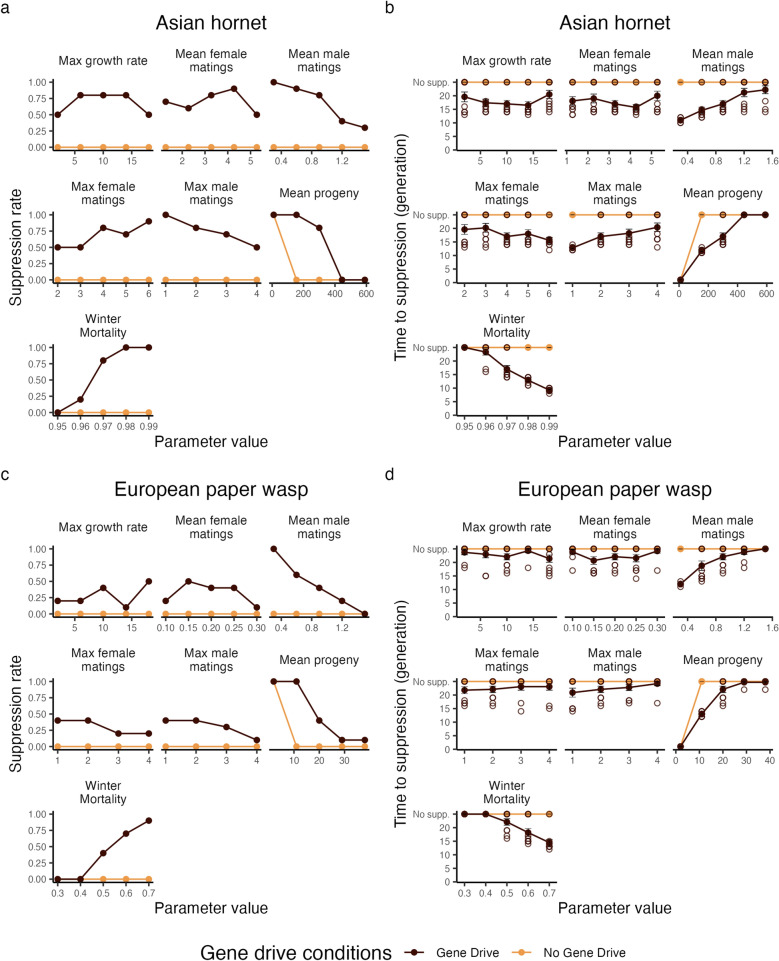
Sensitivity analysis for the biological parameters of the Asian hornet (**a,b**), and the European paper wasp (**c,d**). Panels (**a,c**) show the suppression rate for the gene drive after 25 generations. Panels (**b,d**) show the time required for the gene drive to suppress the population. The open circles indicate the individual repetitions, while the solid circles and the lines show the average time to suppression. The error bars indicate the standard error of the mean. for (**b,d**), no supp. stands for no suppression. In these cases the population was not suppressed after 25 generations.

We see again that the gene drive is more effective in the Asian hornet (Fig. 4a,b) than in the European paper wasp (Fig. 4c,d). Across the whole range of parameters, the suppression rate is higher in the Asian hornet (Fig. 4a), and the time to suppression is lower (Fig. 4b). We mostly see changes in the suppression rate, as opposed to only slight changes in the time to suppression. In both species, mean male matings, max male matings, number of progeny, and winter mortality have a strong effect on the efficacy of the gene drive. In all these cases, except for winter mortality, increasing the parameter leads to a decrease in the efficacy of the gene drive. Changing the max growth rate and the max and mean female matings did not show a clear effect on the suppression rate in either species.

One life history trait that differs between both species we did not fully capture in our sensitivity analysis is polyandry, which is a queen mating with multiple males. This trait is a combination of mean female matings and max female matings, an interaction we did not explore as we only looked at first order effects. While the Asian hornet is a highly polyandrous species, the European paper wasp is not. To untangle the effects of polyandry on the success of the gene drive, we modeled the Asian hornet without polyandry, and the European paper wasp with doubled polyandry (Supplementary Fig. S3). We increased the paper wasp mean female mating rate from the default 0.2 to 0.4, and increased the max female matings to 4. For the Asian hornet, we decreased the max female matings to 1. As the distribution of female matings is zero truncated, this effectively brought the mean female matings to 1 as well.

Removing polyandry from the Asian Hornet strongly reduces the efficacy of the gene drive (Supplementary Fig. S3a). In the case of a gene drive targeting female infertility using female release, suppression decreases from 64 to 20 out of 100 repetitions. When using male release for female infertility, suppression did not occur anymore. This is in line with the trend suggested by decreasing the max female matings in Fig. 4a. However, we do not see a clear positive effect of adding polyandry to the European paper wasp on the efficacy of the gene drive (Supplementary Fig. S3b). Suppression increases marginally from 20 to to 24 out of 100 replicates for a gene drive targeting female fertility using female release. Comparing these results to the sensitivity analysis (Fig. 4c), this strongly suggests this is only due to noise, meaning there was no effect of adding polyandry to the European paper wasp. The suppression rate does not change and remains 6 when using the same gene drive with male release results.

## Discussion

Invasive social hymenopterans, such as the Asian hornet, can cause significant ecological and commercial damage in their invasive ranges. Difficulties in the application of conventional methods for the control of invasive social hymenopterans have led to a surge of interest in the possibility of using gene drive to suppress such invasive populations. In this paper we have modelled the efficacy of gene drives for population suppression of two invasive social wasps, the Asian hornet (*Vespa velutina nigrithorax)* and the European paper wasp (*Polistes dominula*) under a variety of gene drive and life history parameters. We also find that a gene drive targeting fertility is only effective when targeting females. When a gene drive targeted male fertility or the fertility of both sexes, it could not spread. We also find that the high sexual productivity and life history of social insect colonies represent a significant limiting factor for the efficacy of gene drives. These factors produce hard limits on drive technical efficiency, below which a gene drive was never able to effectively suppress modelled populations. Overall, our results indicate that, despite the potential value of gene drive as a control agent, such methods will only be effective in suppressing invasive social insect populations if driving alleles are able to achieve high levels of technical efficiency.

We showed that although a gene drive can suppress a social wasp population, it can only do so under fairly stringent gene drive-specific conditions. This is the case for two reasons: firstly, high per-colony surviving offspring numbers allow resistance allele to arise in the population, thus limiting the success of suppression gene drives. Due to the reversal of longevity/fecundity trade-offs, reproductive females in social hymenopteran species may reach levels of fecundity magnitudes higher than those possible for non-social organisms^65,66^. Notably, even though mortality rates are likely to be extremely high for Asian hornet females in the wild (over 0.95^56,67^), successful Asian hornet nests are extremely productive (~300 gynes/nest), such that the number of sexual females per group that survived to found new nests each year was both high and consistent across the two species we modeled. This fecundity reduces the effects of drift and therefore the potential efficacy of gene drive: the more surviving progeny each gene drive carrier female produces, the more likely that all possible allele combinations will be represented among those progeny, thereby reducing the likelihood that any given allele will be fixed by chance. For the same reason, the probability that a novel resistance allele will be lost by drift before being spread by positive selection is reduced when female fecundity is higher. This effect is somewhat counteracted by the overwintering mortality, which can significantly reduce the amount of offspring that survives to reproduce. Consequently, species with either a lower number of offspring, or higher winter mortality will require a less stringent gene drive efficiency. Intriguingly, there appears to be a fine balance of colony productivity to overwintering mortality in the social hymenopterans that we modeled: increasing or decreasing either of these parameters strongly affected the likelihood that an introduced drive allele would be able to successfully suppress a given population (Fig. 4).

In our model, four factors increase this effect of random drift, and thus show a strong effect on gene drive efficacy: number of offspring, winter mortality, mean male mating, and max male mating. Besides the clear effects of number of offspring and winter mortality, male mating rates are important because when male mating rates are low, a larger part of the male population will remain unmated as the male mating rate is not zero-truncated. This will make males a limiting factor to fertilise females, thus effectively reducing the total reproductive output of the population. This effect does not hold true for the female mating rates, because their mating rate distributions are zero-truncated, as we assume that females will always find a mate if one is available.

The effect of polyandry is less clear, though present. Especially when we remove polyandry from the Asian hornet, we see a strong decrease in suppression rate. We did not find a clear increase in suppression rate in the European paper wasp when increasing its polyandry however. This is likely due to the fact that there already was very little polyandry to begin with, so doubling it might not have had a strong effect. Taken together, these results suggest that polyandry is likely to decrease gene drive efficacy by reducing random drift. We do not observe an increase in the speed of spread of the gene drive as previously reported^64^. This difference is likely due to the fact that we divide the sperm over the amount of offspring instead of increasing the number of offspring of a queen after polyandry. In a naturally occurring gene drive in mice, polyandry was found to confer resistance against the spread of this gene drive^68^. This natural gene drive in mice confers a fertility cost by damaging non-gene drive sperm. This shows that if a gene drive confers any type of fitness or fertility cost, polyandry could be a mechanism through which resistance forms. Further mating complexities could provide additional mechanisms, such as mate choice and spatial mating networks^64^. Polyandry varies considerably in social hymenopterans, even within genera^69^, and understanding the exact nature of its effect on the spread of a gene drive will be important to properly apply gene drives to social hymenopteran suppression.

We did not observe appreciable reductions in population size in response to the introduction of a gene drive in our modelled populations, except in those cases where complete suppression occurred (Supplementary Fig. S1). This apparent implasticity of population size is the result of a combination of features of our model. First, because per-nest gyne productivity greatly exceeded rates of winter mortality, wasps have a great reproductive potential. Due to inefficiencies in the gene drive, there are always enough fertile queens to sustain the population at carrying capacity. For example, when the gene drive is at very high frequency, say 0.48 (Fig. 2) (0.5 being the highest frequency possible), in a population of 1000 Asian hornets, this would mean that there are 960 wt/gd queens and 40 wt/wt queens. These 40 queens would already give 40 $\times$ 2 $\times$ 300 $\times$ (1 − 0.97) = 720 wt alleles to the next generation of queens (number of queens $\times$ queen’s wt alleles $\times$ number of offspring $\times$ (1 − winter mortality)). Additionally, with a cutting rate of 0.95, the wt/gd queens would pass 960 $\times$ (1 − 0.95) $\times$ 300 $\times$ (1 − 0.97) = 432 wt alleles to the next generation of queens (number of queens $\times$ (1 − cutting rate) $\times$ number of offspring $\times$ (1 − winter mortality)). These same numbers hold for the drones the queens produce, resulting in there always being enough wildtype alleles and, thus, enough fertile queens to keep the population at carrying capacity. So overall, the gene drive at default values is too inefficient to impact the population size, except in those cases where it reaches such high levels that the population is suppressed entirely.

Second, we assume that infertile gynes are incapable of effectively competing with wildtype gynes and therefore do not contribute to density-dependent mortality. This is rooted in our assumption that early-season nests founded by infertile queens will fail quickly due to such females’ inability to produce workers. For this reason, in our models, infertile gynes are removed from the population before density-dependent mortality occurs, and fertile individuals experience a lower density-dependent mortality, thus preventing a reduction in population size. It is likely that, if density-dependent mortality were applied prior to the removal of infertile females, we would observe significantly stronger reductions in population size in response to the introduction of driving alleles in our models. The importance of density dependence in gene drive models has been discussed in Dhole et al.^59^, and our models of social hymenopterans confirm that the ecology of a species is critical for gene drive dynamics and outcomes.

A notable feature of the apparently high productivity to mortality ratio of invasive social wasps is that this ratio appears to place hard minimum limits on the technical efficiency required for a drive allele to successfully spread. For example, we found that complete suppression of hornet populations was close to impossible for a driving allele whose probability of non-homologous end-joining exceeded ~0.15, even when other parameters (such as the probability of functional repair) were set to extremely efficient values (Fig. 3). Importantly, while many technical gene drive parameters may be improved with continued research, the probability of non-homologous end-joining appears to be a largely biologically intrinsic factor that varies considerably between taxa^19–22,24^. As such, the upper limit for this parameter indicated by our models suggests that gene drives may vary strongly in their suitability across different social hymenopteran taxa.

Our results further indicate that, in social wasps, a driving element that targets fertility can only successfully spread if its effects are female-specific. Gene drives targeting fertility either in males or in both sexes were never successful in our modelling, because haploid males are unable to act as asymptomatic carriers of the gene drive. Our results therefore indicate that any successful attempt to control invasive haplodiploid species using fitness-targeting gene drives will necessarily target females rather than males, in line with the findings of a previous modeling study^39^. This fact may represent a significant impediment to the biological control of invasive haplodiploids using gene drives, since the rate of formation of resistance alleles is expected to be substantially higher in females than in males^70^.

We found that a neutral gene drive could spread much more reliably than one targeting fertility. In theory, a gene drive without any directly detrimental phenotypic effects can still contribute to biocontrol, if the gene drive leaves affected individuals vulnerable to further control measures. For example, a gene drive that disrupts resistance to a specific pesticide could allow that pesticide to be used as a control measure once the gene drive has reached high frequency or become fixed^40^. However, such an approach requires targeted management techniques, which are not currently available for invasive social hymenopterans excepting a few ant species^71,72^. As such, direct targeting of female fertility is likely to remain the most promising route for managing invasive social hymenopterans using gene drive.

Despite the challenges presented by haplodiploidy and high fecundity, we found that a gene drive targeting female fertility could spread to a significant frequency (just under 0.5) within social wasp populations before either of two outcomes happens: a resistance allele forms and rescues the population, or by chance no fertile individuals were produced or no fertile queens successfully mated. Combined with the usual generation of resistance alleles in a short number of generations, this result indicates that a homing gene drive would be ineffective at suppressing invasive social wasp populations or at best, unreliably effective.

High productivity and haplodiploidy represent two significant challenges to the potential efficacy of gene drive as a control agent for invasive social wasps. A third challenge is the risk that the gene drive could spread to other wasp species by hybridization: rates of inter-specific hybridization appear to be significantly higher among hymenopterans than other arthropods^73^, and the introgression of a driving allele into native wasp species is a meaningful risk given that social wasps perform important ecosystem services in their native ranges^74^. Thus, even if the technical efficiency of drive can be optimised to the point that it is a viable option for the control of invasive wasps, gene drive’s safety as a control agent may remain a significant concern.

Like any model, ours includes assumptions and simplifications. Parameterisation of the model proved difficult due to a relative paucity of life history data for vespid wasps, despite the ecological importance of this group^74^. For example, we assume *prima facie* that female mating rates are zero-truncated, such that females never fail to find at least one male with whom to mate. This assumption may become unrealistic for very small population sizes, but empirical data that would allow us to accurately model this effect are lacking. For this reason, we instead used estimates from several closely related species as described in our methods. Another potential limitation of our model is our assumption that infertile queens do not compete with fertile ones for resources since they do not start a colony. This assumption is conservative, as infertile queens could still compete for resources before colonies are started, for example, early in the year for nest sites. Finally, other limitations of our model include the lack of any spatial component, including a complete lack of immigration and emigration, and the assumption of perfect admixture without mate choice.

In terms of our modelled gene drive, we have assumed parameters according to experimental data as shown in mosquitoes^22,61^. Therefore, we might be overestimating the efficiency of gene drives in this study, as they are less efficient in *Drosophila melanogaster*^20,70,75^ and mice^23,24^. We conclude that for both the Asian hornet as the European paper wasp, a viable gene drive will need to be highly efficient to overcome the challenges posed by the fecundity and ecology of these species. However, the predicted necessary gene drive parameters to suppress a wasp population are near to what has been achieved in mosquitoes, so this scenario is not unthinkable^21,22,61^. Nevertheless, potential further inefficiencies in a gene drive might render them an unrealistic control method for social wasps. Besides the assumptions of our gene drive parameters, a further limitation of our modelling is the relative simplicity of our modelled gene drive mechanism. We model homing in the germline, but ignore any potential embryonic deposition of Cas9 that would lead to additional formation of resistance alleles^70,76^. We also do not model early or late germline resistance allele formation that could happen due to asynchronous Cas9 expression and homology directed repair^24,70,76^. Finally, the gene drive fitness cost is implemented as being fully dominant, whereas the mechanism could be more complex^63^. All in all, we see that a gene drive already has a very limited set of parameters under which it could suppress a population of social wasps. Our conclusion that a gene drive will need to be highly efficient to suppress a hornet population is likely to be exacerbated when taking into account all these potential complications. Thus, experimental tests of a gene drive social wasps are essential to estimate the applicability of gene drives for population suppression.

## Conclusions

We have modeled the spread of a homing gene drive under a variety of conditions of life history and drive efficacy through populations of two invasive social wasps: the Asian hornet and the European paper wasp. We find that, due to large progeny numbers produced by reproductive females in these species, combined with their specific life history, a homing allele can only reach fixation under highly efficient drive conditions. These findings, together with limitations imposed by haplodiploidy and potential for inter-specific hybridization, highlight the difficulty of applying genetic biocontrol measures to social hymenopterans. We conclude that, although the fecundity and life history of social hymenopteran populations imposes hard requirements upon the technical efficiency of drive, it is nonetheless plausible that currently-available gene drive methods will be able to achieve the efficiency required to suppress such populations. With sufficient testing, the gene drive approach modelled here could eventually complement or replace more conventional approaches such as nest destruction and bait trapping.

## Supplementary Information

Supplementary Figures.

## Data Availability

Our model code and data can be found on the HighlanderLab GitHub: https://github.com/HighlanderLab/ymeiborg_hornet_gd.

## References

[1] Mack RN (2000). Biotic invasions: Causes, epidemiology, global consequences, and control. Ecol. Appl..

[2] Seebens H (2017). No saturation in the accumulation of alien species worldwide. Nat. Commun..

[3] Arca M (2015). Reconstructing the invasion and the demographic history of the yellow-legged hornet, *Vespa velutina*, in Europe. Biol. Invas..

[4] Monceau K, Bonnard O, Thiéry D (2014). *Vespa velutina*: A new invasive predator of honeybees in Europe. J. Pest. Sci..

[5] Laurino D, Lioy S, Carisio L, Manino A, Porporato M (2019). *Vespa velutina*: An alien driver of honey bee colony losses. Diversity.

[6] Husemann M, Sterr A, Mack S, Abraham R (2020). The northernmost record of the Asian hornet *Vespa velutina* nigrithorax (Hymenoptera, Vespidae). Evol. Syst..

[7] Villemant C (2011). Predicting the invasion risk by the alien bee-hawking Yellow-legged hornet *Vespa velutina* nigrithorax across Europe and other continents with niche models. Biol. Conserv..

[8] Barbet-Massin M, Salles JM, Courchamp F (2020). The economic cost of control of the invasive yellow-legged Asian hornet. NeoBiota.

[9] Abrol DP (1994). Ecology, behaviour and management of social wasp *Vespa velutina* smith (hymenoptera: Vespidae), attacking honeybee colonies. Korean J. Apicult..

[10] Choi MB, Martin SJ, Lee JW (2012). Distribution, spread, and impact of the invasive hornet *Vespa velutina* in South Korea. J. Asia. Pac. Entomol..

[11] Rome Q (2021). Not just honeybees: Predatory habits of *Vespa velutina* (hymenoptera: Vespidae) in France. Ann. Soc. Entomol. Fr..

[12] Villemant, C. *et al.* Bilan des travaux (mnhn et irbi) sur l’invasion en france de *Vespa velutina*, le frelon asiatique prédateur d’abeilles. In *Proc. Journée Sci. Apicole—11 February* 3–12 (2011).

[13] Monceau K, Maher N, Bonnard O, Thiéry D (2013). Predation pressure dynamics study of the recently introduced honeybee killer *Vespa velutina*: Learning from the enemy. Apidologie.

[14] Bier E (2022). Gene drives gaining speed. Nat. Rev. Genet..

[15] Esvelt KM, Smidler AL, Catteruccia F, Church GM (2014). Concerning RNA-guided gene drives for the alteration of wild populations. Elife.

[16] Champer J, Buchman A, Akbari OS (2016). Cheating evolution: Engineering gene drives to manipulate the fate of wild populations. Nat. Rev. Genet..

[17] McFarlane GR, Whitelaw CBA, Lillico SG (2018). CRISPR-based gene drives for pest control. Trends Biotechnol..

[18] Hay BA, Oberhofer G, Guo M (2021). Engineering the composition and fate of wild populations with gene drive. Annu. Rev. Entomol..

[19] DiCarlo JE, Chavez A, Dietz SL, Esvelt KM, Church GM (2015). Safeguarding CRISPR-Cas9 gene drives in yeast. Nat. Biotechnol..

[20] Gantz VM, Bier E (2015). The mutagenic chain reaction: A method for converting heterozygous to homozygous mutations. Science.

[21] Gantz VM (2015). Highly efficient Cas9-mediated gene drive for population modification of the malaria vector mosquito *Anopheles stephensi*. Proc. Natl. Acad. Sci..

[22] Hammond A (2016). A CRISPR-Cas9 gene drive system targeting female reproduction in the malaria mosquito vector *Anopheles gambiae*. Nat. Biotechnol..

[23] Grunwald HA (2019). Super-Mendelian inheritance mediated by CRISPR–Cas9 in the female mouse germline. Nature.

[24] Pfitzner C (2020). Progress toward zygotic and germline gene drives in mice. CRISPR J..

[25] Esvelt KM, Gemmell NJ (2017). Conservation demands safe gene drive. PLoS Biol..

[26] Noble C (2019). Daisy-chain gene drives for the alteration of local populations. Proc. Natl. Acad. Sci..

[27] Min J, Noble C, Najjar D, Esvelt KM (2017). Daisy quorum drives for the genetic restoration of wild populations. Synth. Biol..

[28] Min J, Noble C, Najjar D, Esvelt KM (2017). Daisyfield gene drive systems harness repeated genomic elements as a generational clock to limit spread. Synth. Biol..

[29] Champer J, Kim IK, Champer SE, Clark AG, Messer PW (2020). Performance analysis of novel toxin-antidote CRISPR gene drive systems. BMC Biol..

[30] Oberhofer G, Ivy T, Hay BA (2019). Cleave and Rescue, a novel selfish genetic element and general strategy for gene drive. Proc. Natl. Acad. Sci..

[31] Dhole S, Lloyd AL, Gould F (2019). Tethered homing gene drives: A new design for spatially restricted population replacement and suppression. Evol. Appl..

[32] Xu X-RS (2020). Active genetic neutralizing elements for halting or deleting gene drives. Mol. Cell.

[33] Wu B, Luo L, Gao XJ (2016). Cas9-triggered chain ablation of cas9 as a gene drive brake. Nat. Biotechnol..

[34] Oh KP (2021). Population genomics of invasive rodents on islands: Genetic consequences of colonization and prospects for localized synthetic gene drive. Evol. Appl..

[35] Wilkins KE, Prowse TA, Cassey P, Thomas PQ, Ross JV (2018). Pest demography critically determines the viability of synthetic gene drives for population control. Math. Biosci..

[36] Lester PJ (2020). The potential for a CRISPR gene drive to eradicate or suppress globally invasive social wasps. Sci. Rep..

[37] Prowse TAA (2017). Dodging silver bullets: Good crispr gene-drive design is critical for eradicating exotic vertebrates. Proc. R. Soc. B Biol. Sci..

[38] Li J (2020). Can crispr gene drive work in pest and beneficial haplodiploid species?. Evol. Appl..

[39] Liu Y, Champer J (2022). Modelling homing suppression gene drive in haplodiploid organisms. Proc. R. Soc. B Biol. Sci..

[40] Faber NR, Meiborg AB, McFarlane GR, Gorjanc G, Harpur BA (2021). A gene drive does not spread easily in populations of the honey bee parasite *Varroa destructor*. Apidologie..

[41] Bertelsmeier C (2021). Globalization and the anthropogenic spread of invasive social insects. Curr. Opin. Insect Sci..

[42] Starks PT, Turillazzi S, West-Eberhard M (2006). Polistes paper wasps: Emergence of a model genus. Ann. Zool. Fenn..

[43] Team, R. C. *R: A Language and Environment for Statistical Computing* (2018).

[44] Gaynor RC, Gorjanc G, Hickey JM (2020). AlphaSimR: An R-package for breeding program simulations. BioRxiv..

[45] Darrouzet E, Gévar J, Guignard Q, Aron S (2015). Production of early diploid males by European colonies of the invasive hornet *Vespa velutina* nigrithorax. PLoS ONE.

[46] Harpur BA, Sobhani M, Zayed A (2013). A review of the consequences of complementary sex determination and diploid male production on mating failures in the Hymenoptera. Entomol. Exp. Appl..

[47] Ranta E, Lummaa V, Kaitala V, Merila J (2000). Spatial dynamics of adaptive sex ratios. Ecol. Lett..

[48] Gardner A, Ross L (2013). Haplodiploidy, sex-ratio adjustment, and eusociality. Am. Nat..

[49] Leadbeater E, Carruthers JM, Green JP, Rosser NS, Field J (2011). Nest inheritance is the missing source of direct fitness in a primitively eusocial insect. Science.

[50] Hughes WOH, Ratnieks FLW, Oldroyd BP (2008). Multiple paternity or multiple queens: Two routes to greater intracolonial genetic diversity in the eusocial Hymenoptera. J. Evol. Biol..

[51] Ding G, Xu H, Oldroyd BP, Gloag RS (2017). Extreme polyandry aids the establishment of invasive populations of a social insect. Heredity.

[52] Ross KG (1983). Laboratory studies of the mating biology of the Eastern Yellowjacket, *Vespula maculifrons* (Hymenoptera: Vespidae). J. Kansas Entomol. Soc..

[53] Wen P (2017). The sex pheromone of a globally invasive honey bee predator, the Asian eusocial hornet, *Vespa velutina*. Sci. Rep..

[54] Southon RJ (2019). High indirect fitness benefits for helpers across the nesting cycle in the tropical paper wasp *Polistes canadensis*. Mol. Ecol..

[55] Strassmann J (2001). The rarity of multiple mating by females in the social Hymenoptera. Insectes Soc..

[56] Archer ME (1984). Life and fertility tables for the wasp species *Vespula vulgaris* and *Dolichovespula sylvestris* (Hymenoptera: Vespidae) in England. Entomol. Generalis.

[57] Dapporto L, Palagi E, Cini A, Turillazzi S (2006). Prehibernating aggregations of *Polistes dominulus*: An occasion to study early dominance assessment in social insects. Naturwissenschaften.

[58] Cervo R, Dapporto L, Beani L, Strassmann J, Turillazzi S (2008). On status badges and quality signals in the paper wasp *Polistes dominulus*: Body size, facial colour patterns and hierarchical rank. Proc. R. Soc. B Biol. Sci..

[59] Dhole S, Lloyd AL, Gould F (2020). Gene drive dynamics in natural populations: The importance of density dependence, space, and sex. Annu. Rev. Ecol. Evol. Syst..

[60] Franklin DN (2017). Invasion dynamics of Asian hornet, *Vespa velutina* (Hymenoptera: Vespidae): A case study of a commune in south-west France. Appl. Entomol. Zool..

[61] Kyrou K (2018). A crispr-cas9 gene drive targeting doublesex causes complete population suppression in caged *Anopheles gambiae* mosquitoes. Nat. Biotechnol..

[62] Champer SE (2020). Computational and experimental performance of crispr homing gene drive strategies with multiplexed grnas. Sci. Adv..

[63] Langmüller AM (2022). Fitness effects of crispr endonucleases in drosophila melanogaster populations. Elife.

[64] Verma P, Reeves RG, Simon S, Otto M, Gokhale CS (2022). The effect of mating complexity on gene drive dynamics. Am. Nat..

[65] Blacher P, Huggins TJ, Bourke AF (2017). Evolution of ageing, costs of reproduction and the fecundity–longevity trade-off in eusocial insects. Proc. R. Soc. B Biol. Sci..

[66] Séguret A, Bernadou A, Paxton RJ (2016). Facultative social insects can provide insights into the reversal of the longevity/fecundity trade-off across the eusocial insects. Curr. Opin. Insect Sci..

[67] Martin SJ (1993). Weight changes in adult hornets, *Vespa affinis* (Hymenoptera: Vespidae). Insectes Soc..

[68] Manser A, König B, Lindholm AK (2020). Polyandry blocks gene drive in a wild house mouse population. Nat. Commun..

[69] Loope KJ, Chien C, Juhl M (2014). Colony size is linked to paternity frequency and paternity skew in yellowjacket wasps and hornets. BMC Evol. Biol..

[70] Champer J (2018). Reducing resistance allele formation in crispr gene drive. Proc. Natl. Acad. Sci..

[71] Sunamura E (2011). Combined use of a synthetic trail pheromone and insecticidal bait provides effective control of an invasive ant. Pest Manag. Sci..

[72] Buczkowski G, Wossler TC (2019). Controlling invasive argentine ants, linepithema humile, in conservation areas using horizontal insecticide transfer. Sci. Rep..

[73] Weyna A, Bourouina L, Galtier N, Romiguier J (2022). Detection of F1 hybrids from single-genome data reveals frequent hybridization in Hymenoptera and particularly ants. Mol. Biol. Evol..

[74] Brock RE, Cini A, Sumner S (2021). Ecosystem services provided by aculeate wasps. Biol. Rev..

[75] Guichard A (2019). Efficient allelic-drive in drosophila. Nat. Commun..

[76] Kandul NP (2020). Assessment of a split homing based gene drive for efficient knockout of multiple genes. G3 Genet. Genes Genomes.

