# Supplementary material for: The suppressive potential of a gene drive in populations of invasive social wasps is currently limited
Source: Sci Rep. 2023 Jan 30;13:1640. doi: 10.1038/s41598-023-28867-8 (PMC9886928; doi:10.1038/s41598-023-28867-8)
Supplement: Supplementary file 1 — Supplementary Figures. [file 41598_2023_28867_MOESM1_ESM.pdf]

The suppressive potential of a gene drive in populations of invasive social wasps is currently limited

Adriaan B. Meiborg[1,2,*], Nicky R. Faber[1,3], Benjamin A. Taylor[4], Brock A. Harpur[4], and Gregor Gorjanc[1]

[1]HighlanderLab, The Roslin Institute and Royal (Dick) School of Veterinary Studies, The University of Edinburgh, Easter Bush Campus, Midlothian EH25 9RG, United Kingdom
[2]Developmental Biology Unit, European Molecular Biology Laboratory, Meyerhofstraße 1, 69117 Heidelberg, Germany
[3]Laboratory of Genetics, Department of Plant Sciences, Wageningen University & Research, Droevendaalsesteeg 1, 6708 PB Wageningen, The Netherlands
[4]Department of Entomology, Purdue University, West Lafayette, IN 47907, United States of America
[*]y.meiborg@gmail.com

Supplementary Material

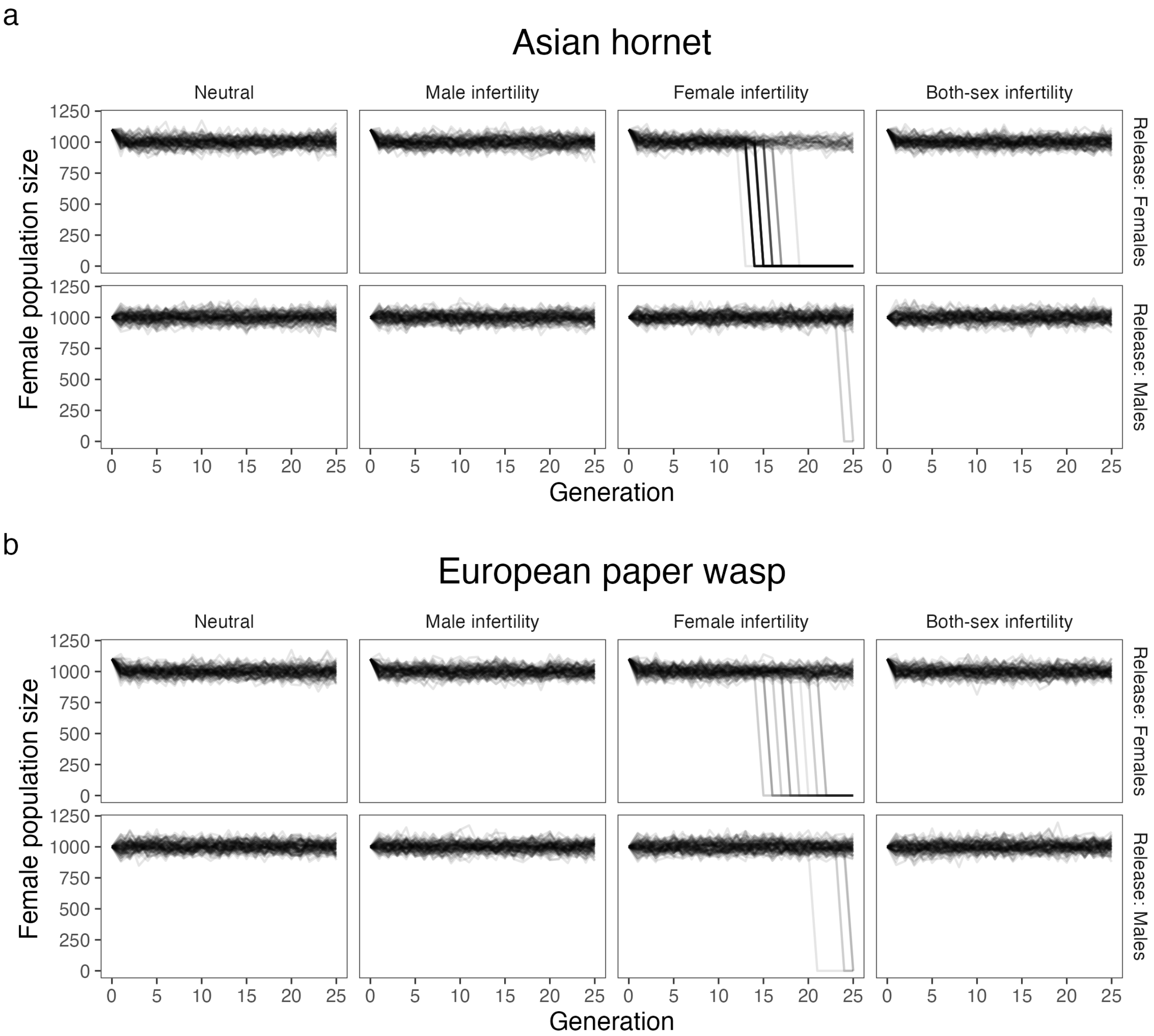

Figure S1. Female population size for Asian hornet (**a**) and European paper wasp (**b**) by gene drive strategy and release carriers (females or males). The different strategies (neutral, male infertility, female infertility, and both-sex infertility) determine how the gene drive operates. In the neutral strategy there is no fitness cost to having the gene drive, whereas in the infertility strategies, the designated sex cannot reproduce when homo- or hemizygous for the gene drive.

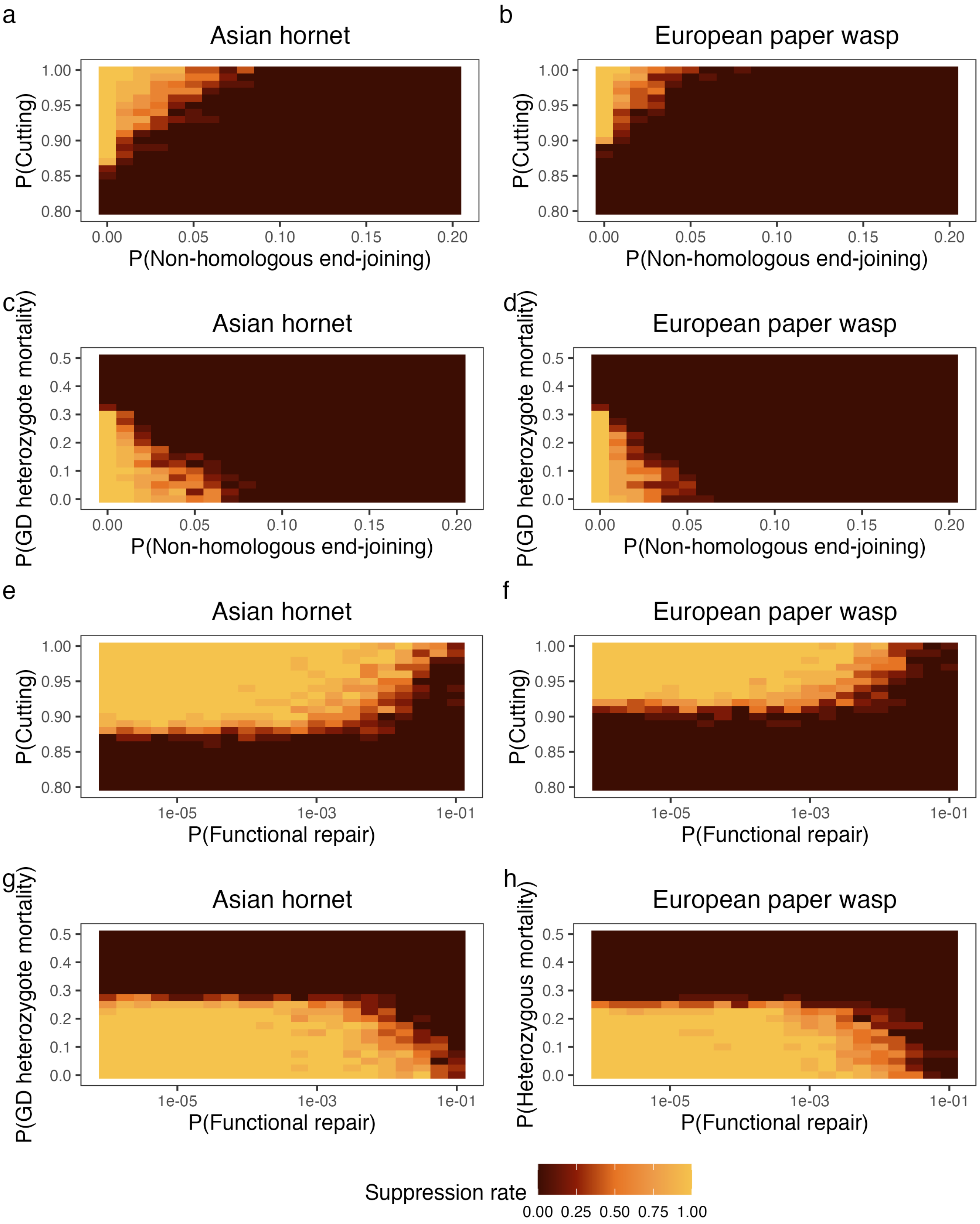

Figure S2. Heatmaps of the suppression rate in the Asian hornet (**a,c,e,g**) and the European paper wasp (**b,d,f,h**) using gene drives that have varying probabilities of functional repair (P_{FR}) and non-homologous end-joining (P_{NHEJ}) against variable rates or cutting (P_{cut}) and mortality of gene drive heterozygotes ($P_{HetMort}$). Note that the model was run for 50 generations instead of 25 to reduce the noise and better allow the different scenarios to resolve.

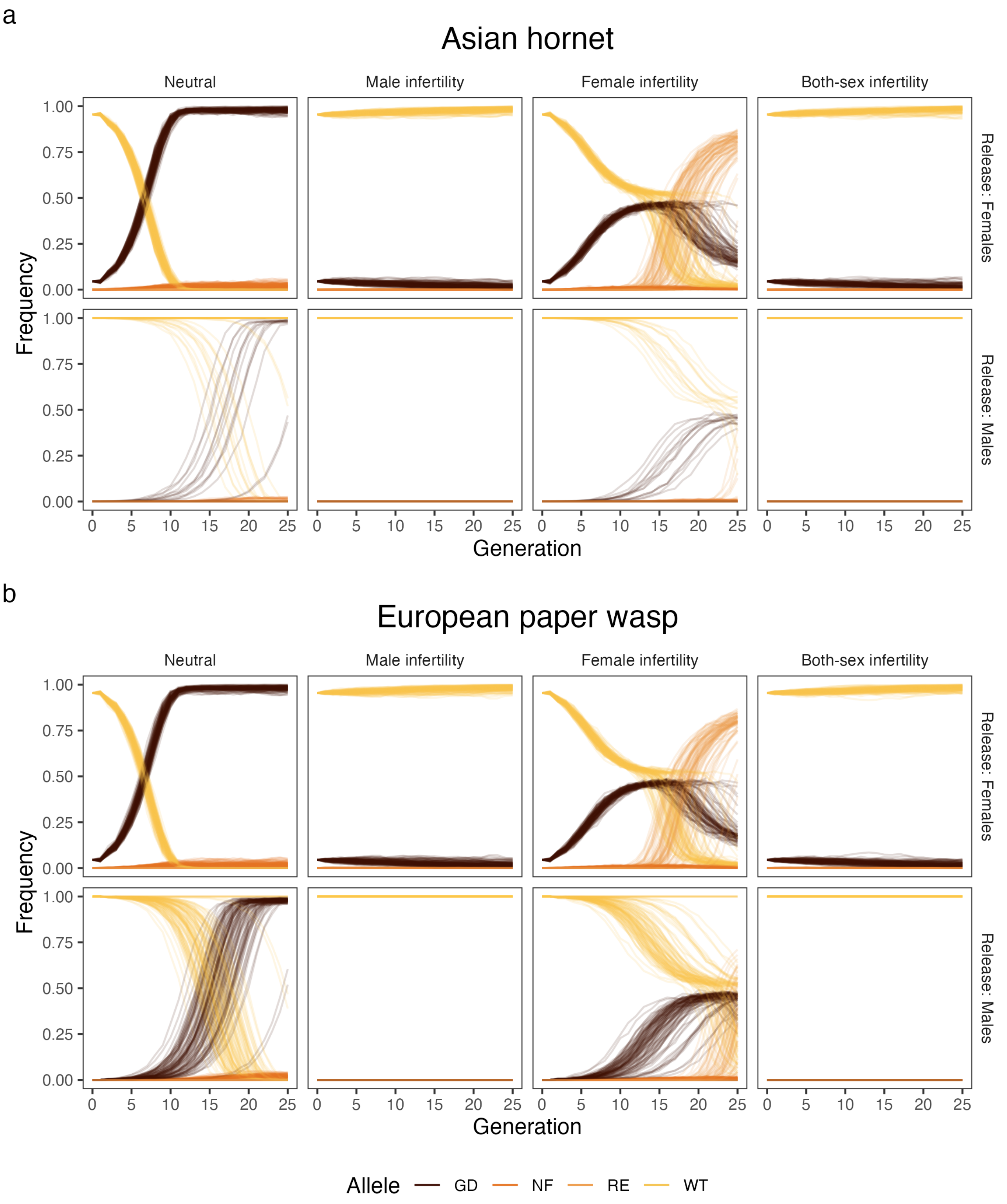

Figure S3. Frequencies of wildtype (WT), gene drive (GD), resistance (RE), and non-functional (NF) alleles in a female Asian hornet population with no polyandry (**a**), and a female European paper wasp population with polyandry (**b**) by gene drive strategy and release carriers (females or males). The different strategies (neutral, male infertility, female infertility, and both-sex infertility) determine how the gene drive operates. In the neutral strategy there is no fitness cost to having the gene drive, whereas in the infertility strategies, the designated sex cannot reproduce when homo- or hemizygous for the gene drive.
